# Hybrid & El Tor variant biotypes of *Vibrio cholerae* O1 in Thailand

**Published:** 2011-04

**Authors:** M. Na-Ubol, P. Srimanote, M. Chongsa-nguan, N. Indrawattana, N. Sookrung, P. Tapchaisri, S. Yamazaki, L. Bodhidatta, B. Eampokalap, H. Kurazono, H. Hayashi, G.B. Nair, Y. Takeda, W. Chaicumpa

**Affiliations:** *Department of Microbiology & Immunology, Faculty of Tropical Medicine, Mahidol University, Bangkok, Thailand*; 1 *Graduate Studies, Faculty of Allied Health Sciences, Thammasat University, Pathumthani, Thailand*; 2 *Office for Research & Development, Faculty of Medicine Siriraj Hospital, Mahidol University, Bangkok, Thailand*; 3 *Department of Veterinary Science, Graduate School of Life & Environmental Sciences, Osaka Prefecture University, Osaka, Japan*; 4 *Department of Enteric Diseases, Armed Force Research Institute of Medical Science, US Army Component, Bangkok, Thailand*; 5 *Bamrasnaradura Institute, Nonthaburi, Thailand*; 6 *Obihiro University of Agriculture & Veterinary Medicine, Department of Animal & Food Hygiene, Hokkaido, Japan*; 7 *Department of Microbiology & Nutrition, Chugoku-gakuen University, Okayama, Japan*; 8 *National Institute of Cholera & Enteric Diseases, Kolkata, India*; 9 *Department of Parasitology & Faculty of Medicine Siriraj Hospital, Mahidol University, Bangkok, Thailand*

**Keywords:** Biotypes, El Tor variant, Thailand, *Vibrio cholerae*

## Abstract

**Background & objectives:**

El Tor *Vibrio cholerae* O1 carrying *ctxB^C^* trait, so-called El Tor variant that causes more severe symptoms than the prototype El Tor strain, first detected in Bangladesh was later shown to have emerged in India in 1992. Subsequently, similar *V. cholerae* strains were isolated in other countries in Asia and Africa. Thus, it was of interest to investigate the characteristics of *V. cholerae* O1 strains isolated chronologically (from 1986 to 2009) in Thailand.

**Methods:**

A total of 330 *V. cholerae* O1 Thailand strains from hospitalized patients with cholera isolated during 1986 to 2009 were subjected to conventional biotyping *i.e*., susceptibility to polymyxin B, chicken erythrocyte agglutination (CCA) and Voges-Proskauer (VP) test. The presence of *ctxA, ctxB, zot, ace, toxR, tcpA^C^, tcpA^E^, hlyA^C^ and hlyA^E^* were examined by PCR. Mismatch amplification mutation assay (MAMA) - and conventional- PCRs were used for differentiating *ctxB* and *rstR* alleles.

**Results:**

All 330 strains carried the El Tor virulence gene signature. Among these, 266 strains were typical El Tor (resistant to 50 units of polymyxin B and positive for CCA and VP test) while 64 had mixed classical and El Tor phenotypes (hybrid biotype). Combined MAMA-PCR and the conventional biotyping methods revealed that 36 strains of 1986-1992 were either typical El Tor, hybrid, El Tor variant or unclassified biotype. The hybrid strains were present during 1986-2004. El Tor variant strains were found in 1992, the same year when the typical El Tor strains disappeared. All 294 strains of 1993-2009 carried *ctxB^C^* ; 237 were El Tor variant and 57 were hybrid.

**Interpretation & conclusions:**

In Thailand, hybrid *V. cholerae* O1 (mixed biotypes), was found since 1986. Circulating strains, however, are predominantly El Tor variant (El Tor biotype with *ctxB^C^*).

*Vibrio cholerae*, the causative agent of severe watery diarrhoeal disease cholera, comprises 206 serogroups (O1-O206) based on antigenic diversity of their outer membrane lipopolysaccharides^1^,^2^. Strains of the O1 serogroup are divided into two biotypes *i.e*., classical and El Tor, according to their phenotypic differences. The classical strains are sensitive to 50 units of polymyxin B and Mukerjee’s type IV bacteriophage while the El Tor strains are generally dually resistant with the exception of some strains isolated in southern Bangladesh^3^,^4^. The El Tor strains are more adapted and resilient in environment, and cause higher infection to case ratio and more asymptomatic carriers than the classical counterpart^5^. Clinical manifestations of cholera caused by classical *V. cholerae* are more severe and prolonged than those caused by the El Tor^6^,^7^. This is attributable to the subtle difference of cholera toxin (CT) encoded by *ctxAB* genes of *V. cholerae*. Each of the *V. cholerae* O1 biotype can be divided into three serotypes *i.e*., Ogawa, Inaba, and Hikojima. Since 1817, the world has experienced seven cholera pandemics caused by *V. cholerae* O1. Strains of classical biotype were considered as the causative agents for the first six pandemics while the 7^th^ cholera pandemic which started in 1961 from Sulawesi Island, Indonesia, was caused by El Tor *V. cholerae* O1. Since then, the El Tor *V. cholerae* had replaced the classical biotype as the sole cause of cholera epidemics until 1982 when there was a re-emergence of the classical *V. cholerae* isolated from patients during an epidemic in Bangladesh^8^–^10^. Both biotypes co-existed in Bangladesh until the classical vibrios became extinct in 1993. Until 1991, only toxigenic *V. cholerae* O1 strains caused cholera epidemic and pandemics. In 1992, a large cholera outbreak was reported from southern India and subsequently spread rapidly to neighbouring countries in several countries in Asia but did not spread to any other continent. The epidemic organism was non-O1 *V. cholerae* which could not be allocated into any of the pre-existing non-O1 serogroups. Subsequently, the organism was designated as serogroup O139 synonym Bengal in recognition of the place of origin^11^–^13^.

New *V. cholerae* O1 variants carrying mixed classical and El Tor phenotypes were first isolated from hospitalized patients with severe watery diarrhoea in Matlab, Bangladesh, in 2002^3^. These isolates could not be allocated into the classical or El Tor biotype using conventional biotyping tests. Genotypically, these were found to carry the El Tor genome backbone including El Tor specific gene clusters: VSP-I and -II and RTX, indicating that these belonged to El Tor lineage. These isolates carried different combinations of alleles of *tcpA* and CTX prophage repressor gene (*rstR*)^4^. Their classical biotype characteristic was due to the presence of the classical CTX prophage and the deduced amino acids of the nucleotide sequence coding for cholera toxin B subunit belonged to classical biotype. Similar strains were isolated in Mozambique in 2004^14^. Subsequently, *V. cholerae* O1 El Tor variants have been reported from several Asian countries including China, Japan, Hong Kong, Sri Lanka, and Vietnam and Africa (Zambia)^15^. In a retrospective study of *V. cholerae* strains isolated in Kolkata, India, during a 17 year period (1989-2005), using mis-match amplification mutation assay (MAMA)-PCR for determining *ctxB* alleles, it was revealed that the El Tor strains carrying *ctxB* allele of the classical biotype (*ctxB^C^*) have emerged since 1991 and co-existed with the prototype El Tor strains until 1995 when these completely replaced the typical El Tor biotype. Arbitrarily, the *V. cholerae* O1 strains carrying mixed phenotypes of classical and El Tor biotypes [polymyxin B (50 units) susceptibility and positive for chicken erythrocyte agglutination (CCA) and Voges-Proskauer (VP) test] are designated hybrid biotype where as the *V. cholerae* O1 with typical El Tor phenotypes (resistant to 50 units of polymyxin B, and positive for CCA and VP test) but carrying *ctxB^C^* are designated El Tor variant^16^. This nomenclature has been followed in this study.

The 7^th^ pandemic cholera arrived in Thailand in 1963, when the El Tor strains completely replaced the classical vibrios and established endemicity^17^. The O139 Bengal was first isolated from hospitalized patient with severe watery diarrhoea in Thailand in 1993^18^. The O139 serogroup completely disappeared from Thailand since 1996^17^. Because it is known that classical *V. cholerae* strains with *ctxB^C^* inflicted more severe symptoms than the typical El Tor infection^6^,^16^ and because there had been a resurgence of cases of severe watery diarrhoea that required hospitalization during 1999-2002, it was of interest to make an insight into both phenotypic and genotypic characteristics of *V. cholerae* O1 isolated from cholera patients in different years in Thailand.

## Material & Methods

*Bacterial strains*: A total of 330 *V. cholerae* O1 strains (248 Ogawa, 82 Inaba) isolated from hospitalized patients with cholera in various regions of Thailand from 1986 to 2009 (Table I) were investigated. Nineteen *V. cholerae* O1 strains collected from Australia, Bangladesh, India, Peru, Romania and Thailand in different years were used as reference strains^4^,^19^ (Table II). Among them, 16 strains were obtained from the collection of the Laboratory Science Division, the International Centre for Diarrhoeal Disease Research of Bangladesh, Dhaka, Bangladesh; two strains (G27875 and SC11) were provided by Dr T. Ramamurthy, the National Centre of Cholera and Enteric Diseases, Kolkata, India; and one strain (295/33) was from the Department of Microbiology and Immunology, Faculty of Tropical Medicine, Mahidol University, Bangkok, Thailand. All strains were subjected to conventional biotyping methods (susceptibility to 50 units of polymyxin B, CCA and VP test)^20^ using strains 569B and N16961 as the classical and El Tor reference strains, respectively.

**Table I:** *V. cholerae* O1 strains isolated from Thailand during 1986-2009

Year of isolation (n)	Strain no.	Serotype	Phenotype			Genotype		Biotype (see also Table IV)	Number of strain(s)/total number of strain(s) of the year
			PB	CCA	VP	*ctxB*	*rstR*		
1986 (5)	1-2	Inaba	R	+	+	E	E	El Tor	2/5
	3	Inaba	R	+	+	E	E+C	El Tor	1/5
	4	Inaba	S	-	+	E	E	Hybrid group 1	1/5
	5	Inaba	R	+	+	E+C	E	Unclassified group 1	1/5
1987 (1)	6	Inaba	R	+	+	E	E	El Tor	1/1
1989 (2)	7	Inaba	R	-	+	E+C	E+C	Hybrid group 2	1/2
	8	Inaba	S	+	+	E	E+C	Hybrid group 3	1/2
1990 (13)	9-12	Inaba	R	+	+	E	E+C	El Tor	4/13
	13-16	Ogawa	R	+	+	E	E	El Tor	4/13
	17-18	Inaba	R	+	+	E+C	E+C	Unclassified group 2	2/13
	19-21	Ogawa	R	+	+	E+C	E	Unclassified group 1	3/13
1991 (4)	22	Ogawa	R	+	+	E	E	El Tor	1/4
	23-25	Ogawa	R	+	+	E+C	E	Unclassified group 1	3/4
1992 (11)	26	Inaba	R	+	+	E	E+C	El Tor	1/11
	27	Inaba	S	+	+	E	E	Hybrid group 4	1/11
	28	Ogawa	R	+	+	E	E	El Tor	1/11
	29	Ogawa	R	+	+	E+C	E	Unclassified group 1	1/11
	30-33	Ogawa	R	+	+	C	E+C	El Tor variant	4/11
	34-36	Ogawa	R	-	+	C	E+C	Hybrid group 5	3/11
1993 (9)	37-38	Inaba	R	+	+	C	E+C	El Tor variant	2/9
	39-43	Ogawa	R	+	+	C	E+C	El Tor variant	5/9
	44	Ogawa	R	+	-	C	E+C	Hybrid group 6	1/9
	45	Ogawa	R	+	+	C	C	El Tor variant	1/9
1994 (7)	46	Inaba	R	+	-	C	E+C	Hybrid group 6	1/7
	47-51	Ogawa	R	+	+	C	E+C	El Tor variant	5/7
	52	Ogawa	S	+	+	C	E+C	Hybrid group 7	1/7
1995 (11)	53-62	Ogawa	R	+	+	C	E+C	El Tor variant	10/11
	63	Ogawa	R	+	-	C	E+C	Hybrid group 6	1/11
1996 (3)	64-65	Ogawa	R	+	+	C	E+C	El Tor variant	2/3
	66	Ogawa	S	+	+	C	E+C	Hybrid group 7	1/3
1997 (3)	67	Ogawa	R	+	+	C	E+C	El Tor variant	1/3
	68-69	Ogawa	R	+	+	C	C	El Tor variant	2/3
1998 (2)	70-71	Ogawa	R	+	+	C	C	El Tor variant	2/2
1999 (179)	72-78	Inaba	R	+	+	C	C	El Tor variant	7/179
	79-83	Ogawa	R	+	+	C	E+C	El Tor variant	5/179
	84-85	Ogawa	R	+	-	C	E+C	Hybrid group 6	2/179
	86-115	Ogawa	R	+	-	C	C	Hybrid group 8	30/179
	116-247	Ogawa	R	+	+	C	C	El Tor variant	132/179
	248	Ogawa	R	-	+	C	C	Hybrid group 9	1/179
	249	Ogawa	R	-	-	C	C	Hybrid group 10	1/179
	250	Ogawa	S	+	+	C	C	Hybrid group 11	1/179
2000 (21)	251-270	Ogawa	R	+	+	C	C	El Tor variant	20/21
	271	Ogawa	R	+	-	C	C	Hybrid group 8	1/21
2001 (27)	272-294	Inaba	R	+	+	C	C	El Tor variant	23/27
	295-298	Inaba	R	+	-	C	C	Hybrid group 8	4/27
2002 (13)	299-306	Inaba	R	+	+	C	C	El Tor variant	8/13
	307	Inaba	R	+	-	C	C	Hybrid group 8	1/13
	308-310	Inaba	S	+	+	C	C	Hybrid group 11	3/13
	311	Ogawa	R	+	+	C	C	El Tor variant	1/13
2003 (8)	312-315	Inaba	R	+	+	C	C	El Tor variant	4/8
	316	Inaba	R	+	-	C	C	Hybrid group 8	1/8
	317	Inaba	S	+	+	C	C	Hybrid group 11	1/8
	318	Inaba	S	+	-	C	C	Hybrid group 12	1/8
	319	Inaba	S	-	+	C	C	Hybrid group 13	1/8
2004 (9)	320-324	Inaba	R	+	+	C	C	El Tor variant	5/9
	325-327	Inaba	R	+	-	C	C	Hybrid group 8	3/9
	328	Inaba	S	+	-	C	C	Hybrid group 12	1/9
2009 (2)	329-330	Ogawa	R	+	+	C	C	El Tor variant	2/2

n, total number of strain(s) of the indicated year; PB, susceptibility to 50 units of polymyxin B; CCA, chicken red blood cell agglutination; VP, Voges-Proskauer test; MAMA, mismatch amplification mutation assay; R, resistant; S, sensitive; +, positive; -, negative; C, classical; E, El Tor

**Table II:** *V. cholerae* O1 reference strains isolated from various countries

No.	Name of isolate (n=19)	Year of isolation	Country of origin	Serotype	Phenotype			Genotype		Biotype	Originally identified biotype
					PB	CCA	VP	*ctxB*	*rstR*		
1	569B	1948	India	Inaba	S	-	-	C	C	Classical	Classical
2	GP71	1971	India	Ogawa	R	+	+	C	E	El Tor variant	El Tor
3	N16961	1975	Bangladesh	Inaba	R	+	+	E	E	El Tor	El Tor
4	2463-78	1978	Australia	Inaba	R	-	-	C	C	Hybrid	El Tor
5	GP156	1979	Australia	Ogawa	R	+	-	C	E	Hybrid	El Tor
6	2164-88	1988	United states	Inaba	R	+	+	C	C	El Tor variant	El Tor
7	295/33	1990	Thailand	Ogawa	R	-	+	E+C	E	Hybrid	El Tor
8	C6706	1991	Peru	Inaba	R	+	+	E+C	E	Hybrid	El Tor
9	C7754	1991	Romania	Ogawa	R	+	-	C	E+C	Hybrid	El Tor
10	MJ1485	1994	Bangladesh	Inaba	R	-	+	C	C	Hybrid	El Tor
11	B33	2004	Mozambique	Ogawa	R	+	-	C	C	Hybrid	El Tor
12	AR15493	Unknown	Bangladesh	Inaba	R	+	+	C	E	El Tor variant	El Tor
13	AR15425	Unknown	Bangladesh	Inaba	R	+	+	C	E	El Tor variant	El Tor
14	G27875	Unknown	India (NICED)	Ogawa	R	+	+	C	E	El Tor variant	El Tor
15	SC11	Unknown	India (NICED)	Ogawa	R	+	+	C	E	El Tor variant	El Tor
16	GP12	Unknown	India	Ogawa	R	+	-	C	E	Hybrid	El Tor
17	AS230	Unknown	India	Ogawa	R	+	+	C	E	El Tor variant	El Tor
18	AS231	Unknown	India	Ogawa	R	+	+	C	E	El Tor variant	El Tor
19	AS233	Unknown	India	Ogawa	R	-	+	C	E	Hybrid	El Tor

PB, susceptibility to 50 units of polymyxin B; CCA, chicken red blood cell agglutination;, VP, Voges-Proskauer test; R, resistant; S, sensitive; +, positive; -, negative; C, classical; E, El Tor

*Conventional- and MAMA-PCRs*: All *V. cholerae* strains were examined for the presence of *ctxA, ctxB, zot, ace, toxR, tcpA^C^, tcpA^E^, hlyA^C^* and *hlyA^E^* by conventional PCR using strains AR15493 and AR15425 from Bangladesh as positive controls for *zot, ace, toxR,* and *hlyA* genes and strain C6706 as positive control for *ctxAB* and *tcpA*^19^. Conventional biotyping methods and a combination of MAMA- and conventional- PCRs were used for classifying the strains into prototype El Tor, hybrid, or El Tor variant biotypes, based on their *ctxB* and *rstR* genes^21^–^23^. Strains MJ1485 from Bangladesh and B33 from Mozambique served as hybrid biotype reference strains while G27875 and SC11 from NICED, India, were El Tor variant reference strains.

Primer sequences used in PCRs are shown in Table III^19^. Amplification mixture (25 μl) for *ctxB*-MAMA-PCR and rstR-PCR composed of 1 μl bacterial genomic DNA template, 2.5 μl 10× PCR buffer, 2 μl each of 2.5 mM deoxynucleotide triphosphate (Fermentas, Vilnius, Lithuania), 2 μl of 25 mM MgCl_2_, 2 μl of 10 μM of individual forward and reverse primers (Bio Basic Inc., Toronto, Canada), 0.5 units *Taq* DNA polymerase (Fermentas) and sterile ultra pure distilled water. Amplification of other genes was essentially the same as described previously^19^. The PCR products were analyzed by using 1.5 per cent agarose (Seakem LE, BMA, Glendate, CA, USA) gel electrophoresis and ethidium bromide staining (Sigma Chemical Co., USA). A Gel Doc 2000 (Bio-Rad, CA, USA) was used for DNA band documentation.

**Table III:** PCR primers for the study of *V. cholerae* O1 genes

Gene (s)	Primer sequence	Size of PCR amplicon (bp)	PCR condition						Reference
			Initial denaturation	Denaturation	Annealing	Extension	Final extension	No. of cycles	
Simple PCR									
*rstR^E^*	Forward: GCACCATGATTTAAGATGCTC	501 (El Tor)	94°C, 5 min	94°C, 60 s	58°C, 60 s	72°C, 90 s	72°C, 7 min	30	22
	Reverse: TCGAGTTGTAATTCATCAAGAGTG								
*rstR^C^*	Forward: CTTCTCATCAGCAAAGCCTCCATC	474 (Classical)	94°C, 5 min	94°C, 60 s	64°C, 60 s	72°C, 90 s	72°C, 7 min	30	22
	Reverse: TCGAGTTGTAATTCATCAAGAGTG								
MAMA-PCR									
*ctxB*	Forward: ACTATCTTCAGCATATGCACATGG		96°C, 2 min	96°C, 10 s	55°C, 10 s	72°C, 30 s	72°C, 2 min	25	21
	Reverse for El Tor: CTGGTACTTCTACTTGAAACA								
	Reverse for classical: CTGGTACTTCTACTTGAAACG								
MAMA-PCR, mismatch amplification mutation assay-PCR								

## Results & Discussion

All of the 330 *V. cholerae* O1 Thai clinical strains collected over 24 years (1986-2009) were found to carry *ctxA, ctxB, zot, ace, toxR, tcpA^E^* and *hlyA^E^* which verified genetically their toxin producing capacity and epidemic potential. Two hundred and sixty six strains were prototype El Tor (resistant to the polymyxin B, and positive for CCA and VP test) and the remaining 64 strains were not biotypable (Table I).

Identification of *rstR* by conventional PCR showed that the 36 strains of 1986-1992 carried either the El Tor *rstR* (*rstR^E/C^*) or combination of the El Tor and classical *rstR* (*rstR^E/C^*) (Table I). MAMA-PCR for ctxB of these isolates revealed that 18 (50%) carried *ctxB^E^*. Only 15 of these 18 strains had prototype El Tor phenotype (resistant to 50 units of polymyxin B, and positive for CCA and VP test) indicating that they were typical El Tor biotype. The other 3 strains, although carrying *ctxB^E^*, appeared to be hybrid biotype as they possessed mixed phenotypes (Table I and IV). There were 11 strains of 1986-1992 (31%) that carried *ctxB^E/C^*. Among these only one strain had mixed classical and El Tor phenotypes implying that this was hybrid biotype. The remaining 10 with *ctxB^E/C^*, however, could not be assigned into any of the redefined biotype scheme^16^ although these showed conventional El Tor phenotype (Tables I and IV. The remaining seven (19%) of the 1986-1992 (all were isolated in 1992) strains carried *ctxB ^C^*; four of these had conventional El Tor phenotypes implying that these were El Tor variant while the other three had mixed phenotypes, and were hybrid (Table I). These data indicate the presence of hybrid biotype of *V. cholerae* O1 in Thailand since 1986 or even before and these co-existed with the typical El Tor strains. The *V. cholerae* O1 Thailand strains that carried *ctxB^E^/rstR^E^*i.e**., typical El Tor strains, were found for the last time in 1992 in this *V. cholerae* O1 collection which was the same year when the strains of El Tor variant biotype (strains 30-33) carrying *ctxB^C^/rstR^E/C^* emerged in the country (Table I). It is noteworthy that in 1992 the epidemic *V. cholerae* O139 strains emerged in Southern India^11^. The Fig. shows MAMA-PCR results of representative strains of *V. cholerae* chronologically isolated in Thailand *i.e*., *ctxB^C^* (Fig. A) and *ctxB^E^* (Fig. B).

**Table IV:** Biotypes of the 330 *V. cholerae* Thailand clinical strains

Biotype	Genotype		Phenotype		
	*ctxB*	*rstR*	PB	CCA	VP
Classical	C	C	S	-	-
El Tor	E	E	R	+	+
El Tor	E	E+C	R	+	+
Hybrid group 1	E	E	S	-	+
Hybrid group 2	E+C	E+C	R	-	+
Hybrid group 3	E	E+C	S	+	+
Hybrid group 4	E	E	S	+	+
Hybrid group 5	C	E+C	R	-	+
Hybrid group 6	C	E+C	R	+	-
Hybrid group 7	C	E+C	S	+	+
Hybrid group 8	C	C	R	+	-
Hybrid group 9	C	C	R	-	+
Hybrid group 10	C	C	R	-	-
Hybrid group 11	C	C	S	+	+
Hybrid group 12	C	C	S	+	-
Hybrid group 13	C	C	S	-	+
El Tor variant	C	C	R	+	+
El Tor variant	C	E+C	R	+	+
Unclassified group 1	E+C	E	R	+	+
Unclassified group 2	E+C	E+C	R	+	+

PB, susceptibility to 50 units of polymyxin B; CCA, chicken red blood cell agglutination; VP, Voges-Proskauer test; R, resistant; S, sensitive; +, positive; -, negative; C, classical; E, El Tor

**Fig.**
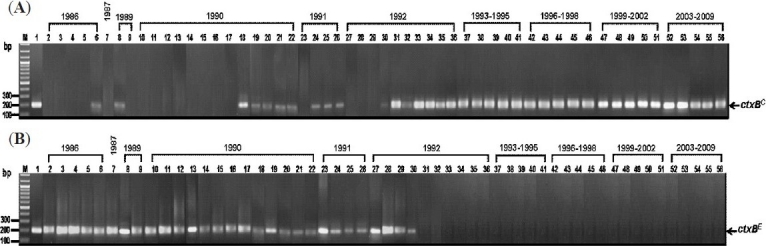
Results of MAMA-PCR for amplification of *ctxB^C^* **(A)** and *ctxB*^E^ **(B)** from representative *V. cholerae* strains isolated in Thailand during 1986-2009. Lanes 2-6, 1986 strains; lane 7, 1987 strains; lanes 8-9, 1989 strains; lanes 10-22, 1990 strains; lanes 23-26, 1991 strains; lanes 27-36, 1992 strains and lanes 37-56, 1993-2009 strains. Lane M, 100 bp DNA marker. Lane 1 in **(A)**, positive control of *ctxB^C^* (569B); lane 1 in **(B)**, positive control of *ctxB^E^* (N16961).

The *V. cholerae* O1 Thailand strains of 1993-2009 (294) were all found to carry ctxBC and either *rstR^C^* or *rstR ^E/C^*. Majority of these strains (237 strains), however, were El Tor variants as their phenotypes were typical El Tor. The minority (57 strains) belonged to hybrid biotype because these had mixed phenotypes of classical and El Tor (Table I). The 1986-2009 Thailand strains with hybrid biotype could be arbitrarily classified into 13 different hybrid groups, 1-13 (Table IV). During 1986-1992, the biotypes of the 36 *V. cholerae* O1 Thailand strains were 15 prototype El Tor, 7 hybrid (groups 1-5), 4 El Tor variant, and 10 unclassified (unclassified groups 1 and 2) (Tables I and IV). The 294 strains of 1993-2009 belonged to hybrid groups 6-13 (57 strains) and El Tor variants (237 strains) (Tables I and IV).

The *V. cholerae* O1 of hybrid biotype was isolated from patients in India in 1991 when typical *V. cholerae* classical and El Tor biotypes co-existed suggesting the horizontal CTX prophage exchange between strains of the two principal biotypes in order for the infecting strains to be more adapted to the host hostile intestinal environment^15^ which conformed to the more severe cholera symptoms in the afflicted hosts in the recent years^3^,^22^,^24^. It is noteworthy, however, that the classical *V. cholerae* O1 disappeared from Thailand since 1963^25^ when the 7^th^ cholera pandemic caused by typical El Tor strains first hit the Kingdom’s population. There has been no report on the period of co-existing classical and El Tor strains during 1986-2009 within Thailand. Our finding that the *V. cholerae* hybrid biotype could be detected among strains of 1986 suggested that there might be a re-emergence of the classical *V. cholerae* before or during 1986 or there might be other confounding molecular mechanism(s) in the shifting of the characteristics of *V. cholerae* bacteria in Thailand. The speculations warrant detail investigation. In 1992, the epidemic O139 strains emerged in India concurrent with the finding of El Tor variant in Thailand for the first time in this series of strain collection (Table I). Between 1992 and 1993, the *V. cholerae* O1 strains carrying *ctxB^C^* predominated in Kolkata, India^15^ and Thailand (this study). Thus, there seemed to be incomprehensible event of genetic evolution of the *V. cholerae* yielding strains of mixed traits/phenotypes of the two authentic biotypes during this period. After 1994, isolates of *V. cholerae* O1 in Kolkata, India, seemed to carry only *ctxB^C^*; thus these were El Tor variants or hybrids (no phenotypes were given to define the biotype)^16^. Similarity was found among the Thailand strains of this study, however, two years earlier than the Kolkata’s series. All of the Thai strains after 1992 carried *ctxB^C^* of which 57 (19%) were hybrid biotype and 237 strains (81%) were El Tor variants according to the conventional biotyping method and MAMA- and conventional- PCR determinations. In Punjab and Haryana, northern India, where a re-emergence of classical *V. cholerae* has not been reported, the *V. cholerae* hybrid biotype were also found in 2007 (80% of the isolates)^26^. As has been mentioned earlier, many *V. cholerae* isolates of several other countries in Asia and Africa were also found to be biotype hybrid/El Tor variant^15^ indicating that the El Tor *V. cholerae* bacteria, regardless of the geographical areas, tend to evolve for acquisition of the classical CTX prophage. This phenomenon will have impact, more or less, on the treatment of cholera, public health measures, as well as vaccine development.
